# PLASMA AND SKELETAL MUSCLE METABOLIC PATHWAYS ASSOCIATED WITH WEIGHT LOSS, LOW MUSCLE, OR POOR FUNCTION IN CANCER

**DOI:** 10.1093/geroni/igae098.2822

**Published:** 2024-12-31

**Authors:** Lindsey Anderson, Lu Xia, Haiming Kerr, Peter Wu, Atreya Dash, Sina Gharib, Jose Garcia

**Affiliations:** VA Puget Sound Healthcare System, Seattle, Washington, United States; University of Washington, Seattle, Washington, United States; University of Washington, Seattle, Washington, United States; VA Puget Sound Healthcare System, Seattle, Washington, United States; VA Puget Sound Healthcare System, Seattle, Washington, United States; University of Washington, Seattle, Washington, United States; VA Puget Sound Healthcare System, Seattle, Washington, United States

## Abstract

Cancer cachexia (muscle wasting leading to functional impairment) is experienced by most individuals with cancer and increases with age. There are no approved treatments in the U.S./E.U. because Phase III trials have failed to achieve functional endpoints. Identification of metabolites/metabolic pathways related to poor functional performance may reveal therapeutic targets but metabolomics analyses in cancer cachexia have only examined weight loss and/or muscle mass. We characterized metabolomic perturbations in plasma and abdominal wall skeletal muscle from men (n=75; median age=65) with cancer undergoing laparotomy and tested associations with function (handgrip strength “HGS” and stair climb power “SCP” assessed within two weeks pre-surgery), muscle mass (CT L3: cross-sectional area “CT-CSA”), or weight loss (six months pre-surgery). We hypothesized that metabolomic signatures in plasma and muscle would be associated with poorer HGS and SCP and alterations would be distinguishable from associations with weight loss or lower CT-CSA. Spearman correlations and pathway analyses (global test) were performed using “R.” Benjamini-Hochberg false discovery rate (FDR)< 0.1 indicated significance. Lipid/fatty acid peroxidation/transport/metabolism was disrupted in muscle with lower CT-CSA (FDR≤0.09) and in plasma (FDR≤0.07) and muscle (trend; FDR=0.14) with weight loss. Phenylalanine/tyrosine/tryptophan biosynthesis (p=0.045) and amino/nucleotide sugar metabolism (p=0.018) were disrupted in muscle with worse SCP, but not after adjustment (FDR>0.5). Significant/trending dysregulation was not detected in plasma with worse SCP or in muscle/plasma with worse HGS. Lipid metabolism was associated with cancer-related weight loss and low muscle mass. Future studies should analyze metabolomics in activity/mobility-related muscles to elucidate metabolite/pathway dysregulation in cancer-related functional impairment.

